# Cost and cost-effectiveness of BPaL regimen used in drug-resistant TB treatment in the Philippines

**DOI:** 10.5588/ijtldopen.24.0094

**Published:** 2024-06-01

**Authors:** D. Evans, K. Hirasen, D.J. Casalme, M.T. Gler, A. Gupta, S. Juneja

**Affiliations:** ^1^ Health Economics and Epidemiology Research Office, Faculty of Health Sciences, University of the Witwatersrand, Johannesburg,; 2 Health Economics Division, Faculty of Health Sciences, School of Public Health, University of Cape Town, Cape Town, South Africa;; 3 TB-HIV Innovations and Research Foundation,; 4 De La Salle Medical and Health Sciences Institute (DLSMHSI), Dasmariñas, Cavite, The Philippines,; 5 TB Alliance, New York, NY, USA

**Keywords:** bedaquiline, pretomanid, linezolid, economic evaluation, cost analysis

## Abstract

**BACKGROUND:**

In 2022, the WHO announced that the 6-month BPaL/M regimen should be used for drug-resistant TB (DR-TB). We estimate the patient and provider costs of BPaL compared to current standard-of-care treatment in the Philippines.

**METHODS:**

Patients on BPaL under operational research, or 9–11-month standard short oral regimen (SSOR) and 18–21-month standard long oral regimen (SLOR) under programmatic conditions were interviewed using the WHO cross-sectional TB patient cost tool. Provider costs were assessed through a bottom-up and top-down costing analysis.

**RESULTS:**

Total patient costs per treatment episode were lowest with BPaL (USD518.0) and increased with use of SSOR (USD825.8) and SLOR (USD1,023.0). Total provider costs per successful treatment were lowest with BPaL (USD1,994.5) and increased with SSOR (USD3,121.5) and SLOR (USD10,032.4). Compared to SSOR, BPaL treatment was cost-effective at even the lowest willingness to pay threshold. As expected, SLOR was the costliest and least effective regimen.

**CONCLUSIONS:**

Costs incurred by patients on BPaL were 37% (95% CI 22–56) less than SSOR and 50% (95% CI 32–68) less than SLOR, while providers could save 36% (95% CI 21–56) to 80% (95% CI 64–93) per successful treatment, respectively. The study shows that treatment of DR-TB with BPaL was cost-saving for patients and cost-effective for the health system.

## RÉSUMÉ

**CONTEXTE:**

En 2022, l'OMS a annoncé que le traitement BPaL/M de 6 mois devrait être utilisé pour la TB pharmacorésistante (DR-TB). Nous estimons les coûts du BPaL pour les patients et les prestataires par rapport au traitement standard actuel aux Philippines.

**MÉTHODES:**

Des patients sous BPaL dans le cadre d'une recherche opérationnelle, ou un régime oral court standard de 9 à 11 mois (SSOR, pour l’anglais « standard short oral regimen ») et un régime oral long standard de 18 à 21 mois (SLOR, pour l’anglais « standard long oral regimen ») dans des conditions programmatiques ont été interrogés à l'aide de l'outil transversal de l'OMS sur le coût pour les patients atteints de TB. Les coûts des fournisseurs ont été évalués par une analyse ascendante et descendante des coûts.

**RÉSULTATS:**

Les coûts totaux pour les patients par épisode de traitement étaient les plus bas avec BPaL (518,0 USD) et augmentaient avec l'utilisation de SSOR (825,8 USD) et SLOR (1 023,0 USD). Les coûts totaux des prestataires par traitement réussi étaient les plus bas avec BPaL (1 994,5 USD) et ont augmenté avec SSOR (3 121,5 USD) et SLOR (10 032,4 USD). Comparé à SSOR, le traitement BPaL était rentable même au seuil de volonté de payer le plus bas. Comme prévu, le SLOR était le régime le plus coûteux et le moins efficace.

**CONCLUSIONS:**

Les coûts encourus par les patients sous BPaL étaient inférieurs de 37% (IC à 95% 22–56) à ceux du SSOR et de 50% (IC à 95% 32–68) à ceux du SLOR, tandis que les prestataires pouvaient économiser respectivement 36 % (IC à 95% 21–56) à 80% (IC à 95% 64–93) par traitement réussi. L'étude montre que le traitement de la DR-TB par BPaL a permis de réaliser des économies pour les patients et pour le système de santé.

According to the WHO, approximately 21,000 people in the Philippines were infected with drug-resistant TB (DR-TB) in 2021.^1^ Less than half began treatment, while 60% of these patients initiated therapy on standard 18–20-month longer regimens,^1^ which are known to be less efficacious,^2^ and more expensive than shorter standard of care (SOC) treatment options.^3^ Adding to the country’s public healthcare challenges is an underfunded National TB Program (NTP), which has only 37% of the resources needed.^4^ To this effect, cost-saving and cost-effective TB treatment options are critically needed to reduce economic pressure on the currently strained healthcare system.

A promising and increasingly adopted approach to DR-TB treatment is the 3-drug 6-month all-oral regimen BPaL, consisting of bedaquiline, pretomanid, and linezolid. Evidence in support of this shortened treatment option has been generated from the Nix-TB^5^ and ZeNix^6^ trials which demonstrated treatment success rates of 90% 6-months post-treatment initiation, significantly higher than current SOC DR-TB treatment options. These trials, along with subsequent studies: the TB-PRACTECAL trial,^7^ and interim results from a multi-country cohort study, Leveraging Innovation for Faster Treatment of Tuberculosis (LIFT-TB)^8^ have established both reliable and consistent efficacy and safety profiles of BPaL leading to 2022 WHO recommendations for the programmatic use of the regimen.^9^

However, while efficacy and safety profiles have been well-established, real-world cost-related evidence of BPaL is limited.^10^ This study aimed to estimate the patient and provider costs associated with BPaL compared to the current SOC for the treatment of DR-TB in the Philippines.

## METHODS

### Study context

This study was conducted in coordination with the BPaL operational research (OR) programme in the Philippines to estimate A) patient and B) provider costs of BPaL compared to SOC treatment options currently prescribed for DR-TB in the country. The BPaL OR programme was implemented across the Philippines and enrolled 103 patients on BPaL between May 2021 and January 2023.^11^

### Treatment groups

We enrolled patients in three mutually exclusive treatment groups: Group 1) patients who received BPaL, Group 2) patients who received a 9–11-month standard short oral regimen (SSOR), and Group 3) patients who received an 18–20-month standard long oral regimen (SLOR). Treatment with BPaL was administered under the OR programme,^11^ while treatment with both SSOR and SLOR was administered under programmatic conditions.^12^ Importantly, all TB treatment in the Philippines (including treatment provision through the OR programme) is provided to patients free of charge and accessible only through the public health sector.

### Patient costs

#### Cross-sectional survey design

We used the WHO cross-sectional TB patient cost tool to estimate patient costs incurred during TB treatment.^13^ Eligible patients included those ≥14 years with bacteriologically confirmed pulmonary rifampicin resistant (RR)/multidrug-resistant (MDR) TB or pre-extensively drug-resistant (XDR)/XDR-TB^14^ who initiated and were still receiving care at a participating study site between October 2022 and June 2023 and were in their current phase of treatment for at least 14 days. Study teams at respective sites conveniently sampled patients based on the aforementioned eligibility criteria and interviewed eligible patients once – either during the intensive or the continuation phase of treatment. A quarter of the interviews were conducted in person and the remainder telephonically. Reported costs were extrapolated over the entire course of treatment based on prescribed durations for respective phases across respective regimens. The extrapolations inferred that all patients completed treatment.

#### Patient costs

Collected cost data included 1) direct medical costs (drugs, consultation, radiography, procedures such as ultrasounds and biopsies, laboratory tests and hospitalisation); 2) direct non-medical costs (transportation, accommodation, food, etc. for patients and guardians/household members); and 3) indirect costs (among those employed before diagnosis, opportunity costs of time spent on seeking TB care).

#### Catastrophic costs

Catastrophic costs were defined as the proportion of total treatment costs to annual household income before TB diagnosis exceeding thresholds set at 15%, 20% and 25%:^15^

$$Catastrophic\;costs=\;\frac{Total\;treatment\;costs\;\left(direct+indirect\;costs\right)}{Annual\;household\;income\;prior\;to\;TB\;diagnosis}>Threshold\;\%$$

### Provider costs

#### Bottom-up/top-down and ingredients costing

To evaluate provider costs of BPaL and current SOC treatment options, we conducted a bottom-up/top-down and ingredients-based costing analysis.^16^ The former included a retrospective medical record review at three participating study sites and a facility-level financial record review at one site in the Cavite Province (out-patient government clinic) from which salaries, fixed and/or shared costs were sourced and apportioned. Eligible medical records were collected for patients ≥14 years at treatment initiation with bacteriologically confirmed pulmonary RR/MDR-TB, pre-XDR, or XDR-TB.^14^ Records were reviewed from May 2023 to July 2023 and included patients initiating treatment from March 2021 to November 2022 for BPaL, January 2021 to June 2022 for SSOR and January 2021 to November 2021 for SLOR. These initiation periods were deliberately selected to allow all patients the minimum follow-up time required to complete treatment on respective regimens before the commencement of data collection.

Through the retrospective medical record review, collected patient resources included; the number of client-facing health provider interactions (including observed time in motion data with staff salaries sourced from personal communication with the outpatient government clinic head between June 2023 and October 2023), the number of on-treatment clinic visits (from treatment initiation to assigned outcome) and assigned clinical outcome consistent with standard case definitions^12,17^: completed, cured, loss to follow-up (LTFU), died or treatment failure.

Ingredients and respective quantities used in DR-TB treatment were based on 2022 WHO-recommended guidelines for DR-TB treatment,^18^ and included TB-specific monitoring tests such as smear microscopy, culture and imaging etc.; laboratory tests and procedures (unit costs sourced from personal communication with the Chief Medical Technologist for Operations and Biosafety at De Le Salle University’s Medical Centre Laboratory Department in October 2023), and TB-specific and ancillary drugs (unit costs sourced from Stop TB^19^). While TB-specific drugs are not procured or paid for by the facility, including these costs are important in ascertaining accurate cost estimates that may be used for budgetary planning at the above facility- and national programmatic-level. Total cost of patient resource-use was calculated by multiplying average quantities of resources by respective unit costs.

Equipment present in TB-specific treatment areas within the out-patient government TB clinic were documented, categorised (electronic vs. non-electronic) and discounted based on working life years.^20^ Fixed costs such as rent, electricity, water, maintenance, supplies and security were apportioned using a study-specific allocation factor calculated as the proportion of DR-TB-specific visits (headcount) to total facility visits (headcount) within the initiation period used in the file review.

#### Patient outcomes

A full file review was conducted among patients with favourable treatment outcomes defined as the sum of treatment completed and cured (successful treatment).^12,17^ Among those with unfavourable treatment outcomes (the sum of LTFU, died or treatment failure^12,17^), treatment initiation, outcome dates, and clinic outcomes were sourced from The Integrated TB Information System, an electronic public sector TB register linking all public sector clinics. Total time on treatment was calculated as the difference between these dates and total treatment visits were assumed based on total treatment duration and visit scheduling as prescribed in national guidelines.^12^ Average resource-use per visit among those with favourable outcomes was applied to those with unfavourable outcomes and weighted averages among both sets of patients were used to inform total resource-use and cost.

#### Cost-effectiveness

Average cost-effectiveness ratios (ACERs) were used to estimate the ratio of cost to benefit of each treatment regimen (without the use of a comparator):

$$ACER=\frac{\left(Cost\;A\right)}{\left(Effectiveness\;A\right)}$$

Incremental cost-effectiveness ratios (ICERs) were used to estimate the cost-effectiveness of BPaL, compared to SSOR and SLOR:

$$ICER=\frac{\left(Cost\;A-Cost\;B\right)}{\left(Effectiveness\;A-Effectiveness\;B\right)}$$

We considered ICERs against willingness to pay (WTP) thresholds set at 0.5 (USD1,810), 1.0 (USD3,620) and 1.5 (USD5,430) times the gross domestic product (GDP) per capita^21^ in the Philippines in 2022.^22^

### Cost inflation and conversion

All patient costs were collected in 2022 Philippine peso (PHP) and converted to United States Dollar (USD) using the 2022 average exchange rate (USD1 = PHP54.54).^23^ Provider costs followed a similar methodology aside from laboratory costs which were inflated from 2020 to 2022 PHP prior to conversion and drug costs which were originally sourced in 2022 USD. Analyses were performed using SAS v9.4 (SAS Institute Inc., Cary, NC, USA) and MS Excel (Microsoft, Redmond, WA, USA).

### Statistical analysis and reporting

For both patient and provider cost analyses, study populations and cost data were described using descriptive statistics with mean (standard deviation) or median (95% confidence intervals [CIs]) measures of central tendency, frequencies and percentages as appropriate. Patients enrolled in respective analyses’ study groups were compared using χ^2^ tests for categorical data and analysis of variance or the Kruskal–Wallis test for continuous data.

### Ethics

This study was approved by the Asian Hospital and Medical Center Research Ethics Committee, Alabang, Muntinlupa City, Philippines in August 2022. All patients provided written informed consent to participate in the patient cost survey. No direct contact with patients was required for the retrospective medical record review; however, patients consented to the use of their routine clinic data for research purposes at the initiation of treatment.

## RESULTS

### Patient costs

#### Patient profile

Table 1 gives the demographic and clinical characteristics of the enrolled sample. In total, 100 patients, evenly distributed across treatment groups were interviewed and completed the patient cost survey. This included 32% on BPaL and 34% each on SSOR and SLOR, respectively. Characteristics, including age, sex, employment, HIV status, place of diagnosis, time to treatment initiation and main income earner were similar between groups. Statistically significant differences were observed for patient category where 78.1% and 70.6% of those on BPaL and SSOR, respectively, were classified as relapse (defined as patients who have previously been treated for TB, declared cured or treatment completed at the end of their most recent course of treatment, and are now diagnosed with a recurrent episode of TB) or retreatment after LTFU/failure (defined as patients who have previously been treated for TB and who were declared LTFU/treatment failed at the end of their most recent course of treatment), compared to 52.9% of those on SLOR (*P* ≤ 0.05). In terms of drug susceptibility, 12.5% of those on BPaL were classified as pre-XDR/XDR TB compared to none on SSOR and SLOR (*P* ≤ 0.05).

**Table 1.** Characteristics of participants completing the TB patient cost survey (*n* = 100).

		Treatment group			
		BPaL	SSOR	SLOR	*P*-value
		(*n* = 32)	(*n* = 34)	(*n* = 34)	
Characteristic		*n* (%)	*n* (%)	*n* (%)	
Age, years, mean ± SD		42.7 ± 14.5	40.4 ± 15.4	41.5 ± 13.0	0.85
Sex	Female	12 (37.5)	8 (23.5)	14 (41.2)	0.52
Currently employed	Full/part-time/self-employed*	11 (36.7)	10 (34.5)	11 (35.5)	0.98
HIV status	Positive^†^	2 (6.3)	1 (2.9)	1 (2.9)	0.73
Patient category	New	7 (21.9)	10 (29.4)	16 (47.1)	0.00^‡^
	Relapse/retreatment after LTFU/failure	25 (78.1)	24 (70.6)	18 (52.9)	
Place of diagnosis	Primary health clinic^§^	13 (43.3)	13 (46.4)	9 (29.0)	0.34
Time to treatment initiation, days, mean ± SD^¶^		17.3 ± 17.7	17.3 ± 31.6	17.1 ± 26.3	0.30
Drug susceptibility	RR/MDR-TB	28 (87.5)	34 (100.0)	34 (100.0)	0.01^‡^
	Pre-XDR/XDR-TB	4 (12.5)	0 (0.0)	0 (0.0)	
Time of interview	Intensive/early	12 (37.5)	20 (58.8)	19 (55.9)	0.17
	Continuation/late	20 (62.5)	14 (41.2)	15 (44.1)	
Patient is the main or the joint main income earner		17 (77.3)	17 (77.3)	19 (82.6)	0.88

* Excluding students.

† Positive vs. negative, unknown or refused to answer.

‡ *P* ≤ 0.05.

§ Primary health clinic vs. Hospital.

¶ Defined as the time between diagnosis and TB treatment initiation.

BPaL = bedaquiline, pretomanid and linezolid; SSOR = standard short all-oral regimen; SLOR = standard long all-oral regimen; SD = standard deviation; LTFU = loss to follow-up; RR = rifampicin-resistant; MDR = multidrug-resistant; XDR = extensively drug-resistant.

#### Direct, indirect and total treatment costs

Table 2 shows the total on-treatment patient costs by treatment group and cost category. Direct medical, direct non-medical and indirect costs were lowest among patients on BPaL (USD66.1, USD387.7 and USD96.4, respectively) and increased among those on SSOR (USD299.0, USD398.4 and USD225.5, respectively) and SLOR (USD209.5, USD674.9 and USD277.7, respectively). Total treatment costs were lowest among patients on BPaL (USD518.0) and increased among those on SSOR (USD825.8) and SLOR (USD1,023.0) (*P* ≤ 0.05).

**Table 2.** Direct, indirect and total TB treatment costs stratified by treatment group (USD 2022) (*n* = 100).

Cost type	Cost category	BPaL (*n* = 32)*	SSOR (*n* = 34)	SLOR (*n* = 34)
		mean ± SD	mean ± SD	mean ± SD
		USD	USD	USD
Direct medical costs	Medication	22.7 ± 58.1	12.8 ± 60.3	31.1 ± 124.7
	Consultation	5.8 ± 23.6	0.0 ± 0.0	1.5 ± 7.1
	Radiography/procedures	9.8 ± 51.1	75.2 ± 251.7	78.4 ± 365.0
	Laboratory tests	0.0 ± 0.0	0.0 ± 0.0	110.1^†^
	Hospitalisation	172.6 ± 148.6*^¶^*	2,250.6 ± 1,831.2^#^	318.0 ± 317.3^**^
	Total	66.1 ± 176.7	299.0 ± 834.8	209.5 ± 649.4
Direct non-medical costs	Transportation	145.2 ± 221.0	113.0 ± 123.3	205.1 ± 198.3
	Accommodation	0.0 ± 0.0	2.3 ± 12.8	0.0 ± 0.0
	Food/supplements	135.0 ± 211.6	176.5 ± 274.5	356.5 ± 411.6
	Other (linen, soap, etc.)	7.9 ± 43.3	0.1 ± 0.5	0.0 ± 0.0
	Guardian costs (food, accommodation, transport etc.)	168.6 ± 269.0	139.5 ± 168.3	175.6 ± 212.7
	Total	387.7 ± 485.3	398.4 ± 468.4	674.9 ± 538.0
Indirect costs	Income loss^‡^ (time)	96.4 ± 178.6	225.5 ± 418.4	277.7 ± 319.4
Total treatment costs^§^		518.0 ± 596.6^††^	825.8 ± 1,008.0^††^	1,023.0 ± 855.0^††^

* As the BPaL regimen does not have a defined intensive/continuation phase, we defined an early (>2 weeks but <4 months) and late (≥4 months) phase of treatment to extrapolate costs.

† *n* = 1.

‡ Only among patients employed prior to TB diagnosis using reported personal income /hourly wage and where missing, national minimum hourly wage equivalent to USD1.31/h (human capital approach).

§ Direct medical costs + direct non-medical costs + indirect costs.

¶ *n* = 5.

# *n* = 3.

** *n* = 9.

†† *P* ≤ 0.05.

USD = US dollar; BPaL = bedaquiline, pretomanid and linezolid; SD = standard deviation; SSOR = standard short all-oral regimen; SLOR = standard long all-oral regimen.

#### Catastrophic costs of TB care

Table 3 gives catastrophic costs by treatment group. While annual household income prior to diagnosis was similar between groups, the proportion of patients experiencing catastrophic costs at the 15%, 20% and 25% thresholds were significantly lower among those on BPaL (27.3%, 22.7% and 22.7%) compared to SSOR (63.6%, 54.6% and 50.0%) and SLOR (78.3%, 73.9% and 65.2%) (*P* ≤ 0.05).

**Table 3.** Catastrophic costs of patients accessing TB care (USD 2022) (*n* = 100).

		Treatment group			
		BPaL (*n* = 32)	SSOR (*n* = 34)	SLOR (*n* = 34)	*P*-value
		*n* (%)	*n* (%)	*n* (%)	
Annual household income before TB diagnosis, USD	Mean ± SD	4,792.6 ± 4,149.3	4,331.7 ± 5,228.6	5,538.2 ± 12,387.4	0.43
	Missing	10 (31.3)	12 (35.3)	11 (32.4)	
Catastrophic costs^†^ (threshold %)	15	6 (27.3)	14 (63.6)	18 (78.3)	0.00*
	20	5 (22.7)	12 (54.6)	17 (73.9)	0.00*
	25	5 (22.7)	11 (50.0)	15 (65.2)	0.02*

* *P* ≤ 0.05.

† Only among patients reporting annual household income before TB diagnosis.

USD = United States dollar; BPaL = bedaquiline, pretomanid and linezolid; SSOR = standard short all-oral regimen; SLOR = standard long all-oral regimen; SD = standard deviation.

### Provider costs

#### Patient profile

Table 4 shows the demographic and clinical characteristics of patients included in the retrospective medical record review. In total, 144 patients were included, 32 (22.2%) on BPaL, 87 (60.4%) on SSOR and 25 (17.4%) on SLOR. Age and sex were similar between groups while statistically significant differences were observed for patient category where 81.3% and 88.0% of those on BPaL and SLOR respectively were classified as relapse or retreatment after LTFU/failure, compared to 65.5% on SSOR (*P* ≤ 0.05). All of the patients on BPaL achieved a favourable treatment outcome compared to 57.5% on SSOR and 36.0% on SLOR (*P* ≤ 0.05) and among those with favourable outcomes, total treatment visits were significantly lower for BPaL (8.4 visits compared to SSOR (11.1 visits) and SLOR (17.7 visits) (*P* ≤ 0.05).

**Table 4.** Characteristics of patients included in the retrospective medical record review (*n* = 144).

		Treatment group			
Characteristic	Treatment group	BPaL	SSOR	SLOR	*P*-value
		(*n* = 32)	(*n* = 87)	(*n* = 25)	
		*n* (%)	*n* (%)	*n* (%)	
Age, years, mean ± SD		42.4 ± 14.4	41.9 ± 13.0	46.5 ± 18.2	0.56
Female sex		13 (40.6)	28 (32.2)	12 (48.0)	0.31
Patient category	New	6 (18.9)	30 (34.5)	3 (12.0)	0.00*
	Relapse/retreatment after LTFU/failure	26 (81.3)	57 (65.5)	22 (88.0)	
Clinical outcome	Favourable (cured/completed)	32 (100.0)	50 (57.5)	9 (36.0)	0.00*
Treatment visit type					
All patients	Outpatient visits,^†^ mean ± SD	8.4 ± 2.4	8.4 ± 4.0	10.6 ± 6.7	0.18
Favourable outcomes	Outpatient visits,^‡^ mean ± SD	8.4 ± 2.4	11.1 ± 2.3	17.7 ± 2.0	0.00*

* *P* ≤ 0.05.

† Enumerated directly from the medical record review among patients with a favourable treatment outcome and calculated based on time on treatment for those with unfavourable treatment outcomes (two visits in the first month of treatment and monthly visits thereafter). Data were limited to the three participating study sites to facilitate direct comparison. This only applied to patients on SSOR and SLOR as all patients treated with BPaL achieved favourable treatment outcomes.

‡ Enumerated directly from the medical record review.

BPaL = bedaquiline, pretomanid and linezolid; SSOR = standard short all-oral regimen; SLOR = standard long all-oral regimen; SD = standard deviation; LTFU = loss to follow-up.

#### Cost and cost-effectiveness

Table 5 gives the provider costs of TB treatment, as well as the cost-effectiveness ratios of respective treatment regimens. Cost of BPaL per successful treatment was lowest at USD1,994.5 per patient and increased to USD2,371.2 for SSOR and USD5,992.3 for SLOR. The ACER was lowest for treatment with BPaL (USD1,994.5) and increased for treatment with SSOR (USD3,121.5) and SLOR (USD10,032.4). The total cost of BPaL per patient was USD1,994.5 compared to USD1,794.9 for SSOR and USD3,611.7 for SLOR, however, at recognised WTP thresholds of 0.5 (USD1,810), 1.0 (USD3,620) and 1.5 (USD5,430) times GDP per capita, treatment with BPaL was more cost-effective than SSOR (ICER: USD469.4 <WTP thresholds specified) while SLOR costs more and is less effective (dominated; eliminated from consideration strategies).^24^

**Table 5.** Provider costs and cost-effectiveness of TB treatment provision stratified by outcome and treatment group (USD 2022 95% CI).

	Treatment group					
	All outcomes (*n* = 144)			Favourable outcomes (*n* = 91)		
Cost category	BPaL	SSOR	SLOR	BPaL	SSOR	SLOR
	(*n* = 32)*	(*n* = 87)	(*n* = 25)	(*n* = 32)	(*n* = 50)	(*n* = 9)
	Mean USD (95% CI)	Mean USD (95% CI)	Mean USD (95% CI)	Mean USD (95% CI)	Mean USD (95% CI)	Mean USD (95% CI)
TB monitoring	414.1 (413.4–414.8)	436.4 (435.8–437.0)	738.0 (736.5–739.5)	414.1 (413.4–414.8)	612.2 (611.8–612.6)	1,224.4 (1,223.6–1,225.2)
Client-facing providers	42.4 (41.7–43.1)	18.1 (17.5–18.7)	25.4 (23.9–26.9)	42.4 (41.7–43.1)	24.0 (23.6–24.4)	42.1 (41.3–42.9)
Drugs (TB)	568.3 (567.6–569.0)	408.3 (407.7–408.9)	1,686.3 (1,684.8–1,687.8)	568.3 (567.6–569.0)	539.4 (539.0–539.8)	2,797.8 (2,797.0–2,798.6)
Drugs (non-TB)	391.6 (390.9–392.3)	322.6 (322.0–323.2)	347.6 (346.1–349.1)	391.6 (390.9–392.3)	426.1 (425.7–426.5)	576.7 (575.9–577.5)
Laboratory tests/procedures	256.9 (256.2–257.6)	263.9 (263.3–264.5)	409.2 (407.7–410.7)	256.9 (256.2–257.6)	348.7 (348.3–349.1)	679.0 (678.2–679.8)
Fixed costs/overhead	320.0 (319.3–320.7)	317.3 (316.7–317.9)	403.8 (402.3–405.3)	320.0 (319.3–320.7)	419.3 (418.9–419.7)	669.9 (669.1–670.7)
Equipment	1.2 (0.5–1.9)	1.2 (0.6–1.8)	1.5 (0.0–3.0)	1.17 (0.5–1.9)	1.5 (1.1–1.9)	2.4 (1.6–3.2)
Total cost per patient, mean (95% CI)	1,994.5 (1,993.8–1,995.2)	1,794.9 (1,794.3–1,795.5)	3,611.7 (3,610.2–3,613.2)	1,994.5 (1,993.8–1,995.2)	2,371.2 (2,370.8–2,371.6)	5,992.3 (5,991.5–5,993.1)
Cost-effectiveness						
ACER^†^	1,994.5	3,121.5	10,032.4			
ICER^‡^	—	469.4	Dominated^§^			

* 100% of patients on BPaL (32/32) completed the full-course of treatment and achieved a favourable treatment outcome.

† Average cost-effectiveness ratio; equivalent to average treatment costs as 100% of patients on BPaL achieved a favourable treatment outcome.

‡ Incremental cost-effectiveness ratio.

§ In incremental cost-effectiveness analysis dominated means to eliminate from consideration strategies (costs more and is less effective)

USD = United States dollar; CI = confidence interval; BPaL = bedaquiline, pretomanid and linezolid; SSOR = standard short all-oral regimen; SLOR = standard long all-oral regimen; ACER = average cost-effectiveness ratio; ICER = incremental cost-effectiveness ratio.

## DISCUSSION

We conducted a cross-sectional patient cost survey and bottom-up micro-/top-down and ingredients costing analysis to estimate the costs of DR-TB treatment in the Philippines. Specifically, we aimed to estimate and compare the costs of the 6-month BPaL regimen to current SOC treatment options.

Costs incurred by patients on BPaL were 37% (95% CI 22**–**56) less than SSOR and 50% (95% CI 32–68) less than SLOR. Importantly, treatment with SSOR and SLOR was administered according to national DR-TB guidelines^12^ and patient costs reported under these regimens are likely true reflections of the current patient experience. In contrast, BPaL was administered under OR protocols,^11^ warranting closer monitoring and more frequent treatment visits.^11^ Associated medication, consultation, transportation and guardian costs (accompanying family/household member) incurred by patients treated with BPaL may then be an overestimation and may likely decrease in routine care, consequently increasing the patient-centred cost-saving benefit of the regimen.

As household income before TB diagnosis did not differ significantly between treatment groups, rates of catastrophic costs reported here may be a direct consequence of total treatment costs. At every threshold (15%, 20%, 25%), patients on BPaL (27.3%, 22.7% and 22.7%, respectively) experienced significantly lower rates of catastrophic costs compared to those treated with SSOR (63.6%, 54.6% and 50.0%, respectively) and SLOR (78.3%, 73.9% and 65.2%, respectively). While a notable improvement, all regimens still fall short of the End TB Strategy target of zero patients facing catastrophic costs due to TB,^25^ leaving room for financial risk protection and social support initiatives for patients but at a reduced scale. It is important to note, however, that when used under programmatic conditions, treatment with BPaL is likely to result in lower patient costs leading to lower proportions of patients facing catastrophic costs than what we report here.

From the provider perspective, at the participating study sites, 100% of patients on BPaL had a favourable treatment outcome (sum of cured and treatment completed) compared to 58% and 36% of patients on SSOR and SLOR respectively. The average cost per patient successfully treated was lowest for BPaL (USD1,994.5) and increased by 19% for SSOR (USD2,371.2) and 200% for SLOR (USD5,992.3). When compared to both SOC treatment options, BPaL was the most cost-effective treatment regimen. Moreover, recent price reductions of up to 55% for key drugs such as bedaquiline^26^ and 34% for pretomanid,^27^ will further reduce the cost and increase the cost-effectiveness of BPaL. On the contrary, more controlled operational research settings under which BPaL was administered may have contributed to high rates of favourable treatment outcomes. If the efficacy of BPaL is lower in routine programmatic care, cost-effectiveness of the regimen may decrease.

### Limitations

The well-known limitations of cross-sectional surveys^13^ apply to our patient cost results and include; variations in interview procedures and recall period which may have resulted in recall bias and inaccurate accounts of direct and indirect costs, the use of a single survey response per patient limited to one of the two potential treatment phases, the extrapolation of costs across a given treatment phase and episode limiting the nuances of the patient experience and associated expenses and the underlying assumption that all patients complete the full course of treatment. To estimate total provider costs, recommended quantities of resources for patients achieving favourable treatment outcomes were applied to those with unfavourable outcomes (TB monitoring tests, TB-specific and ancillary drugs, laboratory tests, and procedures). This may have underestimated the cost to treat patients on SSOR and SLOR as prior to reaching the endpoint of an unfavourable outcome, patients in poorer health may engage with the health system more frequently, requiring more specialised and costlier care. Similarly, costs reported here do not account for the additional resources required to manage adverse events/drug reactions. Thereafter, costs reported here, specifically for treatment with SSOR and SLOR may be an underestimation and the cost-effectiveness of BPaL in relation to these standard regimens may in fact be greater.

## CONCLUSION

In this study, treatment of DR-TB with the WHO recommended 6-month all oral BPaL regimen was cost-saving for patients and cost-effective for providers. These benefits may likely increase when treatment moves from operation research settings to routine care.
